# Association between enteral nutrition support and neurological outcome in patients with acute intracranial haemorrhage: A retrospective cohort study

**DOI:** 10.1038/s41598-019-53100-w

**Published:** 2019-11-11

**Authors:** Xuping Cheng, Weizhe Ru, Kailei Du, Xuandong Jiang, Yongxia Hu, Weimin Zhang, Yingting Xu, Yanfei Shen

**Affiliations:** 1 Department of Intensive Care, Dongyang People’s Hospital, Dongyang, Zhejiang, 322100 P.R. China; 2 Department of Oncology, Cixi People’s Hospital, Cixi, Zhejiang, 315300 P.R. China; 3 Department of Intensive Care Unit, Zhejiang Hospital, Hangzhou, Zhejiang, P.R. China

**Keywords:** Nutrition disorders, Neurological disorders

## Abstract

Association between the amount of enteral nutrition (EN) caloric intake and Glasgow coma scale scores at discharge (GCS_dis_) in intracranial haemorrhage (ICH) was retrospectively investigated in 230 patients in a single center from 2015 and 2017. GCS_dis_ was used as a dichotomous outcome (≤8 or >8: 56/230 vs. 174/230) and its association with the amount of EN caloric intake within 48 hours was analysed in four logistic models. Model 1 used EN as a continuous variable and showed association with favourable GCS_dis_ (odds ratio [OR], 1.04; 95% confidence interval [CI], 1.01–1.08). Models 2 and 3 categorized EN into two (≤25 and >25 kcal/kg/48 hrs) and three caloric intake levels (≤10, 10~25, and >25 kcal/kg/48 hrs) respectively, and compared them with the lowest level; highest EN level associated with favourable GCS_dis_ in both model 2 (OR, 2.77; 95%CI, 1.25–6.13) and 3 (OR, 4.68; 95%CI, 1.61–13.61). Model 4 transformed EN into four quartiles (Q1-Q4). Compared to Q1, OR increased stepwise from Q2 (OR 1.80, 95%CI 0.59–5.44) to Q4 (OR 4.71, 95%CI 1.49–14.80). Propensity score matching analysis of 69 matched pairs demonstrated consistent findings. In the early stage of ICH, increased EN was associated with favourable GCS_dis_.

## Introduction

Intracranial haemorrhage (ICH) remains a significant cause of morbidity and mortality worldwide, and results in significant social burden^1,2^. Nutrition support plays an important role in the intensive care of ICH patients^3^, and enteral nutrition (EN) is the preferred feeding method to maintain gastrointestinal integrity and to prevent translocation of enteric bacteria^4^. Because hypermetabolic responses are almost inevitable following brain injury^5^, studies have indicated that early initiation of EN in ICH patients improve outcomes^6^, such as attenuation of the hypercatabolic response and reduced infection risk. However, the appropriate amount of EN calorie intake for patients in the early stage of ICH remains unclear.

Although hypocaloric feeding has been associated with decreased infection rates^7^ and mortality^8^ in critically ill patients, contrasting reports state that hypocaloric feeding of critically ill patients in the first 7 days of intensive care was associated with a higher incidence of nosocomial infection^9^. However, these inconsistencies in findings may be due to the significant heterogeneity among different cohorts, which makes these results inapplicable to ICH patients.

Thus, we performed this retrospective study to investigate the association between the amount of EN caloric intake and neurological outcome in patients with ICH.

## Results

### Baseline characteristics

Data of 230 patients with ICH were included in this study. In all, 24.3% (56/230) patients had a unfavourable outcome (GCS_dis_ ≤ 8) and the overall in-hospital mortality was 11.3% (26/230). Patients in the high Glasgow coma scale scores at discharge (GCS_dis_) group (9–15) were significantly younger (59.9 ± 14.4 years vs. 51.6 ± 14.9 years, p < 0.001). Further, the amount of EN caloric intake was significantly higher in patients with high GCS_dis_ scores than in those with low GCS_dis_ scores (15.8 ± 13.4 kcal/kg/48 hrs vs. 22.1 ± 13.8 kcal/kg/48 hrs, p = 0.002). The baseline serum albumin level was comparable between the two groups. Detailed comparisons of demographic characteristics are presented in Table 1. The comparisons between two EN intake groups (≤25 and >25 kcal/kg/48 h) are presented in eTable 1.

**Table 1:** Clinical characteristics between two GCS levels.

Variables	3 ≤ GCS ≤ 8 (n = 56)	9 ≤ GCS ≤ 15 (n = 174)	Overall (n = 230)	p
Age (years)	59.9 ± 14.4	51.6 ± 14.9	53.6 ± 15.2	<0.001
Body weight (kg)	62.5 ± 10.2	63.9 ± 9.7	63.6 ± 9.8	0.344
Male [n (%)]	32 (60.3)	114 (65.5)	146 (63.4)	0.258
**Bleeding sites**				
Basal ganglia [n (%)]	13(24.5)	42 (24.1)	55 (23.9)	0.888
Frontal lobe [n (%)]	17 (30.3)	50 (28.7)	67 (29.1)	0.054
Parietal lobe [n (%)]	11 (19.6)	28 (16.1)	39 (16.9)	0.538
Temporal lobe [n (%)]	22 (39.2)	59 (33.9)	81 (35.2)	0.464
Occipital lobe [n (%)]	2 (3.5)	6 (3.4)	8 (3.4)	0.965
Epencephalon [n (%)]	2 (3.5)	7 (4.0)	9 (3.9)	0.880
Epidural hemorrhage [n (%)]	3 (5.3)	24 (13.7)	27 (11.7)	0.088
Subdural hemorrhage [n (%)]	16 (28.5)	39 (22.4)	55 (23.9)	0.347
**Surgery types**				
Intracranial hematoma evacuation [n (%)]	39 (69.6)	131 (75.2)	170 (74.3)	0.403
Decompressive craniectomy [n (%)]	30 (43.4)	65 (41.5)	95 (41.3)	0.094
Craniocerebral drilling and drainage [n (%)]	12 (21.4)	36 (20.6)	48 (20.8)	0.906
**Blood loss**				
Blood loss during surgery (ml)	235.0 ± 197.3	233.7 ± 278.7	234.0 ± 260.8	0.974
**Comorbidities**				
Hypertension [n (%)]	34 (60.7)	79 (45.4)	113 (49.1)	0.046
Diabetes mellitus [n (%)]	4 (7.1)	10 (5.7)	14 (6.0)	0.704
Lung disease [n (%)]	1 (1.7)	15 (8.6)	16 (6.9)	0.080
Liver disease [n (%)]	1 (1.7)	23 (13.2)	24(10.4)	0.015
Alcohol consumption [n (%)]	7 (12.5)	40 (22.9)	47 (20.4)	0.090
**Biochemical indexes**				
White blood cell count (*10^9/L)	13.7 ± 5.3	12.2 ± 4.2	12.6 ± 4.6	0.032
Platelet count (*10^9/L)	156.3 ± 66.5	170.6 ± 59.1	167.1 ± 61.2	0.130
Hemoglobin level (g/L)	107.2 ± 28.0	118.7 ± 94.6	115.9 ± 83.5	0.371
Oxygen partial pressure (mmHg)	165.6 ± 66.1	168.7 ± 55.7	167.9 ± 58.3	0.725
Serum creatinine (mmol/L)	47.9 ± 71.3	42.3 ± 61.6	43.6 ± 63.6	0.563
Serum albumin (g/L)	38.8 ± 42.7	35.5 ± 22.6	36.3 ± 28.5	0.458
Serum sodium (mmol/L)	139.4 ± 4.6	137.7 ± 10.9	137.7 ± 9.8	0.129
**Fluid records**				
Enteral nutrition (kcal/kg/48hrs)	15.8 ± 13.4	22.1 ± 13.8	20.6 ± 13.9	0.002
Fluid intake (ml/kg/48hrs)	116.1 ± 53.8	100.7 ± 34.8	104.5 ± 40.6	0.013
Fluid balance (ml/kg/48hrs)	15.2 ± 42.5	4.7 ± 30.6	7.2 ± 33.6	0.040
**Disease severity scores**				
APACHE II score on admission [median (IQR)]	23.5 ± 5.3	17.6 ± 5.2	19.1 ± 5.8	<0.001
GCS on admission [median (IQR)]	5 (4–7)	8.5 (7–11)	7 (5–10)	<0.001
GCS at discharge [median (IQR)]	5 (3–8)	13 (11–14)	12 (9–14)	<0.001
Clinical outcomes				
Hospital-acquired pneumonia [n (%)]	31 (55.3)	84 (48.2)	115 (50.0)	0.357
Other infections [n (%)]	4 (7.1)	11 (6.3)	15 (6.5)	0.825
Length of ICU stay (days)	13.7 ± 10.5	8.7 ± 7.7	9.9 ± 8.7	<0.001
Length of hospital stay (days)	17.9 ± 11.7	19.9 ± 6.8	19.4 ± 8.3	0.108
In-hospital mortality [n (%)]	26 (46.4)	0 (0)	26 (11.3)	<0.001

Abbreviations: GCS: Glasgow coma scale; APACHE II: acute physiology and chronic health evaluation II; ICU intensive care unit; IQR, interquartile range;

### Association between EN and GCS_dis_

**Figure 1:**
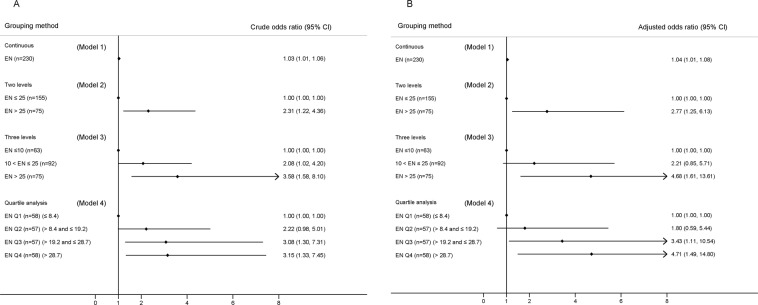
Crude (panel A) and adjusted (panel B) odds ratio of the EN caloric intake in four logistic models. In model 1, EN was used as a continuous variable (1 kcal/kg/48hrs). In model 2, model 3 and model 4, EN was divided into two levels (≤25 and >25 kcal/kg/48 hrs), three levels (≤10, 10~25, and >25 kcal/kg/48 hrs) and four quartiles, respectively, and the lowest level was used as reference level. Abbreviation: EN enteral nutrition.

To test the robustness of the association between EN and GCS_dis_, four logistic models were developed using different methods to stratify the amount of EN caloric intake (Fig. 1, Four logistic models). Model 1 used EN as a continuous variable, and increased EN (1 kcal/kg/48 h) associated significantly with favourable GCS_dis_ (odds ratio [OR], 1.04; 95% confidence interval [CI], 1.01–1.08; p = 0.003). Model 2 classified EN into two levels (≤25 and > 25 kcal/kg/48 h) and high EN levels associated significantly with favourable GCS_dis_ scores (OR, 2.77; 95% CI, 1.25–6.13; p = 0.012). Model 3 classified EN into three levels (≤10, 10–25, >25 kcal/kg/48 h). After adjusting for confounders, only the OR of the highest EN level showed significant association (OR 4.68, 95% CI 1.61–13.61, p = 0.005), compared to the lowest level. Model 4 categorized EN into four quartiles (Q1-Q4). Compared with Q1, OR increased stepwise from Q2 (OR, 1.80; 95% CI, 0.59–5.44; p = 0.294) to Q4 (OR, 4.71; 95% CI, 1.49–14.80; p = 0.008).

### Outcomes after propensity score matching (PSM)

**Figure 2:**
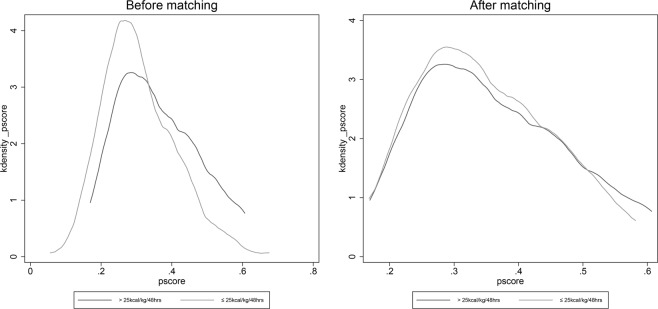
Kernel density plots of p-score before and after propensity score matching.

Patients were divided into two groups according to the amount of EN caloric intake (≤25 and >25 kcal/kg/48 h), and PSM was performed to minimize the imbalance between the groups. After PSM, 69 matched pairs were obtained. Comparisons of the standardized difference of the means, ratio of variances (Table 2), and propensity scores for the two groups (Fig. 2) showed excellent matching among all pairs. All ten variables used for PSM were comparable between the two groups, including the GCS score at intensive care unit (ICU) admission (8 [5–11] vs. 7 [6–10], p = 0.649) and disease severity (Acute Physiology and Chronic Health Evaluation II [APACHE II] score, 18.4 ± 6.1 vs. 18.9 ± 5.2; p = 0.666). Among the matched pairs, the proportion of patients with GCS score >8 was significantly higher in the high EN caloric intake group (60/69 vs. 48/69, p = 0.013), and moreover, the duration of hospital stay was longer (21.5 ± 8.7 vs. 17.8 ± 7.2, p = 0.008), compared to the low EN caloric intake group. Furthermore, hospital-acquired pneumonia tended to be less frequent in the low EN caloric group (27/69 vs. 36/69, p = 0.124).

**Table 2:** Comparisons of confounders after propensity score matching.

Variables	EN ≤25 kcal/kg/48 hrs (n = 69)	EN >25 kcal/kg/48 hrs (n = 69)	p
Age (years)	54.9 ± 16.2	53.3 ± 14.6	0.546
Body weight (kg)	61.9 ± 9.2	62.4 ± 10.1	0.745
Blood loss during surgery (ml)	229.9 ± 184.0	262.1 ± 379.2	0.526
Hypertension [n (%)]	32 (46.3)	32 (46.3)	1.000
GCS on ICU admission [median (IQR)]	8 (5–11)	7 (6–10)	0.649
APACHE II score	18.4 ± 6.1	18.9 ± 5.2	0.666
White blood cell (10^9/L)	12.4 ± 4.3	12.7 ± 4.8	0.686
Platelet count (10^9/L)	158.7 ± 59.7	167.7 ± 60.0	0.379
Serum creatinine level (mmol/L)	48.0 ± 87.5	47.3 ± 35.2	0.949
Serum albumin level (g/L)	34.2 ± 4.3	32.9 ± 4.5	0.093
**Bleeding sites**			
Basal ganglia [n (%)]	17 (24.6)	18 (26.0)	0.854
Frontal lobe [n (%)]	21 (30.4)	27 (39.1)	0.284
Parietal lobe [n (%)]	11 (15.9)	11 (15.9)	1.000
Temporal lobe [n (%)]	22 (31.8)	30 (43.4)	0.160
Occipital lobe [n (%)]	3 (4.3)	3 (4.3)	1.000
Epencephalon [n (%)]	5 (7.2)	1 (1.4)	0.095
Epidural hemorrhage [n (%)]	7 (10.1)	13 (18.8)	0.147
Subdural hemorrhage [n (%)]	14 (20.2)	15 (21.7)	0.834
Blood loss during surgery (ml)	229.9 ± 184.0	262.1 ± 379.2	0.526
**Clinical outcomes**			
GCS at hospital discharge > 8 [n (%)]	48 (69.5)	60 (86.9)	0.013
Length of ICU stay (days)	9.5 ± 8.9	11.5 ± 10.1	0.240
Length of hospital stay (days)	17.8 ± 7.2	21.5 ± 8.7	0.008
Hospital-acquired pneumonia [n (%)]	27 (39.1)	36 (52.1)	0.124
In-hospital mortality [n (%)]	9 (13.0)	2 (2.90)	0.028

Abbreviations: GCS: Glasgow coma scale; ICU intensive care unit; APACHE II: acute physiology and chronic health evaluation II; IQR, interquartile range;

Note: The bleeding sites are not included in the propensity score matching analysis.

## Discussion

In this retrospective study, we found that in ICH patient with relatively low EN caloric intake, an increase in the amount of EN caloric intake was significantly associated with favourable GCS score at discharge (≤25 vs. >25 kcal/kg/48 hrs). The robustness of this finding was verified using different EN grouping methods and PSM analysis. However, because of the retrospective study design, the causal relationship could not be inferred. Therefore, further randomized controlled trials are needed to validate our findings.

Hypermetabolic response^5^ and gastrointestinal dysfunction are common risks factors for malnutrition in ICH patients, which, consequently, is associated with high morbidity and mortality^10^. According to the current consensus^11^, EN is the preferred method to maintain lean body tissue and to preserve the intestinal integrity and reduce the translocation of enteric bacteria^12^. However, the optimal amount of EN caloric intake needed to achieve these outcomes remains unclear.

Several studies have indicated that even with adequate nutrition supply, an acute hypercatabolic status cannot been completely prevented^13,14^. Thus, although malnutrition should be prevented in critically ill patients, evidence supporting a high calorie regimen for ICH patients remains weak.

In the last few decades, studies have investigated the effect of low EN calorie regimen in many diseases. A randomized trial^8^ comprising 240 critically ill patients demonstrated that hypocaloric regimen (60% VS. 90% of the targeted goal), as compared with target feeding, was associated with lower mortality. Yaseen *et al*.^15^, in an observational study of 523 patients, found that a near target caloric intake was associated with significantly higher hospital mortality, ICU-acquired infections, and longer durations of mechanical ventilation and hospital stay. Several underlying causes have been proposed for these findings, such as reduction in the metabolic rate and oxidative stress^16^, generation of mitochondrial free radical^17^, and activation of the plasma membrane redox system^18^.

However, other studies have reported contrasting results. Recent multicentre trials that compared normal EN caloric support showed that the mortality^19,20^ or incidence of infectious complication^20^ were not reduced in the low EN caloric group in critically ill patients. Petros *et al*.^9^ reported that as compared with normal caloric feeding (100% of daily energy expenditure), low caloric feeding (50% of the daily energy expenditure) of critically ill patients during the first 7 days was associated with more incidences of nosocomial infections. In an observational study of 58 patients with poor-grade subarachnoid haemorrhage (SAH), Badjatia *et al*.^21^ found that negative energy balance during the first 7 days results in a potential risk of infections. The inconsistencies among findings may be attributed to both heterogeneity among the different cohorts and different regimens of low caloric intake. In the current study, we found that non-SAH ICH patients who received a high amount of EN caloric intake (>25 kcal/kg/48 hrs) demonstrated better neurological outcomes at discharge, and this finding remained stable both after adjusting for confounders, including GCS scores at emergency admission, and in PSM analysis. In 2015, Badjatia *et al*.^22^ also revealed that in SAH patients, lower caloric intake (<11.3 kcal/kg/day) was associated with a higher risk of infection and negative nitrogen balance, which resulted in a significant risk for a poor neurological outcome. Furthermore, Young *et al*.^23^ showed that compared with patients receiving inadequate gastric feeding, those receiving adequate parenteral nutrition support had a better neurologic outcome at 3 months (mean cumulative caloric balance: 75.6% vs. 59%). However, the early application of parenteral nutrition makes these conclusions inapplicable to ICH patients according to the current guideline^11^. Furthermore, despite named the “high EN caloric group” in the current study, the amount of EN caloric intake in our study was much lower than that in Young *et al*.’s study, which compared to an intervention trial, may be more in line with clinical practice. On the contrary, negative energy balance is associated with increasing number of complications, particularly infections^24^. However, in contrast to previous findings^9,25^ including those in ASH^21,22^, the incidence of hospital acquired pneumonia, a critical complication in ICH patients, was similar in low and high EN caloric groups in the current study. We believe that low EN caloric intake may be a reason for this non-significant finding. The fact that only 32.6% of the included patients received more than 50% of the target EN goal (50 kcal/kg/48 h) in the current study is concerning. Based on our experience, the low EN caloric intake may be attributable to gastrointestinal dysfunction, such as delayed gastric emptying, increased resting energy expenditure, catabolism, immobilization, and delayed and inadequate nutritional supply^11,26^. However, if our findings are validated in future rigorous randomized trials, strategies aiming to improve EN support, such as those involving jejunal feeding and gastrointestinal motility drugs, should be more actively applied for patients in the early stage of ICH.

Our study has several limitations. First, the definition of a high EN calorie regimen was inaccurate as the overall EN caloric intake was relatively low in our cohort. Thus, in contrast to other studies^8,9^, patients receiving>25 kcal/kg/48 h were assigned to the high EN caloric group in the current study. The comparison between the actual low and high EN caloric regimen (for instance, 20% vs. 90% of the target goal) needs further investigation. Furthermore, caloric intake from parenteral nutrition was not included in the current study. In our hospital, the basic fluid protocol for ICH patients is the same, and thus, 5% dextrose and propofol are the main resources of parenteral nutrition. However, the use of both dextrose and propofol is often suspended owing to different conditions, such as fluid overload and sedative status. Thus, calories from parenteral intake were excluded in this analysis. Third, the reason for choosing 2 days as the cut-off point was empirical and somewhat arbitrary. According to clinical experience, the volume of EN intake was largely different in the early stage of ICH patients, while similar in the late stage, and similar finding was also reported in an observational study^3^. Fourth, information on EN-related complications such as diarrhoea, abdominal distension, and vomiting were not available in the electronic medical record system, and thus were not evaluated in the current study. Fifth, although the use of PSM supports our hypotheses, the limitations inherent to the retrospective nature of this study cannot be excluded. Thus, the causal relationship and underlying mechanisms for accelerating neurologic recovery could not be inferred.

## Conclusion

In the early stage of ICH, increased amount of EN caloric intake is associated with improved neurological outcome at hospital discharge, with no significant increase in pneumonia incidence. Future larger randomized clinical trials are required to confirm and validate this association.

## Materials and Methods

### Study location and population

Between June 2015 and September 2017, all patients admitted to the ICU after intracerebral surgery due to ICH in Dongyang People’s Hospital who met the following inclusion criteria were enrolled: (1) Glasgow coma scale (GCS) score ≥4 and ≤12 on admission; (2) Hospital stay duration of more than 3 days; and (3) stable haemodynamics. Patients were excluded if any of the following criteria were met: (1) Age lower than 18 years; (2) Pregnancy; (3) A diagnosis of aneurysmal subarachnoid haemorrhage; (4) Without severe kidney or liver dysfunction; (5) Without uncontrolled hyperglycaemia; and (6) Severe abdominal injury. The general surgery procedures were provided in the Supplementary File. No specific interventions were made, and all patients received standard treatment according to the current guidelines^10,11^. The requirement for informed consent was waived by the ethics committee due to the retrospective nature of the study. The ethics committee of Dongyang People’s Hospital approved this study.

### Data source and primary outcome

All data were extracted from electronic medical records. All demographic characteristics, such as age, sex, weight, and comorbidities, were recorded. Biochemical indexes at ICU admission, including findings from routine blood examinations and clinical scores such as the Acute Physiology and Chronic Health Evaluation II (APACHE II) score, were also extracted. Caloric intake through enteral nutrition within 2 days after admission was calculated. The GCS score at hospital discharge (GCS_dis_) was used as the primary outcome and as a dichotomous outcome in the logistic models: level one: 3–8 and level two: 9–15. For patients who died during the hospital stay, the GCS_dis_ was recorded as 3. The duration of ICU and hospital stay and the occurrence of hospital-acquired pneumonia were recorded as secondary outcomes.

### Grouping methods for EN in logistic regression models

EN was used as a continuous variable in the initial analysis. To test the robustness of the conclusion, EN was further divided into two (≤25 and >25 kcal/kg/48 h) and three levels (≤10, 10–25 and >25 kcal/kg/48 h). For better interpretation, quartile grouping was also applied, and EN quartile 1 was used as the reference in the multivariate logistic regression models.

### Propensity score matching (PSM)

Patients were divided into two groups according to the EN caloric intake (≤25 and >25 kcal/kg/48 h). To minimize the effect of confounding factors such as GCS score at admission, PSM^12^ was applied using a one-to-one nearest neighbour matching algorithm and a calliper of 0.05. The following variables were selected to generate the propensity score: age, body weight, blood loss, hypertension, GCS score at ICU admission, APACHE II score, white blood cell and platelet counts, and serum creatinine and albumin levels. Kernel density plots of the p-score were used to examine the degree of PSM. Matching quality was evaluated by comparing the standardized difference of the means and the ratio of the variances as well as by graphically inspecting the propensity scores for the two groups. Finally, 69 matched pairs were obtained and analysed further.

### Statistical analysis

Continuous variables are expressed as the mean ± standard deviation or median (interquartile range), as appropriate. The student’s t-test or Wilcoxon rank-sum test was used, as appropriate. Categorical data are expressed as proportions, and were compared using the χ^2^ test or Fisher’s exact test. Fourteen confounders with a p value <0.20 in the univariate analyses were included in multivariate logistic regression analyses: age, alcohol consumption, hypertension, frontal lobe and epidural bleeding, thalamencephalon bleeding, platelet and white blood cell count, serum sodium level, lung and liver disease, fluid balance, GCS at emergency department and APACHE II score. A stepwise backward method with p < 0.1 was used to build the model and seven confounders were excluded, including alcohol consumption, hypertension, frontal lobe and epidural bleeding, thalamencephalon bleeding, lung disease, and fluid balance. Multicollinearity was tested using the variance inflation factor (VIF) method, and platelet count, serum sodium level and APACHE II score were excluded for VIF ≥ 5. Four models were included in the final model: age, liver disease, white blood cell count, and GCS at emergency department. PSM was used to minimize between-group imbalances. Two-tailed tests were performed, and p < 0.05 was considered statistically significant. All statistical analyses were performed using Stata 11.2 (Stata Corp., College Station, TX, USA).

## Supplementary information

supplementary file

## Data Availability

All the data are available from the corresponding author on reasonable request.
