# Supplementary material for: Association between enteral nutrition support and neurological outcome in patients with acute intracranial haemorrhage: A retrospective cohort study
Source: Sci Rep. 2019 Nov 11;9:16507. doi: 10.1038/s41598-019-53100-w (PMC6848122; doi:10.1038/s41598-019-53100-w)
Supplement: Supplementary file 1 — supplementary file [file 41598_2019_53100_MOESM1_ESM.docx]

Title page

Association between enteral nutrition support and neurological outcome in patients with acute intracranial haemorrhage: A retrospective cohort study

Xuping Cheng MD.^1^, Weizhe Ru MD.^2^, Kailei Du MD.^1^, Xuandong Jiang MM.^1^, Yongxia Hu MD.^1^, Weimin Zhang MD.^1^, Yingting Xu MM.^1^, Yanfei Shen MD.^3*^

1. Department of Intensive Care, Dongyang People's Hospital, Dongyang, Zhejiang, 322100, P.R. China.
2. Department of Oncology, Cixi People's Hospital, Cixi, Zhejiang, 315300, P.R. China.
3. Department of Intensive Care Unit, Zhejiang Hospital, Hangzhou, Zhejiang, P.R. China.

* Correspondence should be addressed to Yanfei Shen.

E-mail: snow.shen@hotmail.com

Phone: +86-137-3800-9563

eTable 1 Clinical characteristics between two EN categories

| Variables | ≤25 kcal/kg/48 h  (n = 155) | >25 kcal/kg/48 h  (n = 75) | Overall  (n = 230) | p |
| --- | --- | --- | --- | --- |
| Age (years) | 53.8 ± 15.6 | 53.8 ± 14.4 | 53.6 ± 15.2 | 0.906 |
| Body weight (kg) | 64.6 ± 9.4 | 61.5 ± 10.3 | 63.6 ± 9.8 | 0.027 |
| Male [n (%)] | 101 (60.3) | 45 (65.5) | 146 (63.4) | 0.446 |
| Bleeding sites | | | | |
| Basal ganglia [n (%)] | 34(24.5) | 21 (24.1) | 55 (23.9) | 0.312 |
| Frontal lobe [n (%)] | 39 (30.3) | 28 (28.7) | 67 (29.1) | 0.057 |
| Parietal lobe [n (%)] | 27 (19.6) | 12 (16.1) | 39 (16.9) | 0.788 |
| Temporal lobe [n (%)] | 48 (39.2) | 33 (33.9) | 81 (35.2) | 0.052 |
| Occipital lobe [n (%)] | 5 (3.5) | 3 (3.4) | 8 (3.4) | 0.764 |
| Epencephalon [n (%)] | 8 (3.5) | 1 (4.0) | 9 (3.9) | 0.160 |
| Epidural hemorrhage [n (%)] | 14 (5.3) | 13 (13.7) | 27 (11.7) | 0.067 |
| Subdural hemorrhage [n (%)] | 37 (28.5) | 28 (22.4) | 55 (23.9) | 0.983 |
| Blood loss |  |  |  |  |
| Blood loss during surgery (ml) | 226.1 ± 190.4 | 250.2 ± 366.9 | 234.0 ± 260.8 | 0.512 |
| Comorbidities | | | | |
| Hypertension [n (%)] | 77 (60.7) | 36 (45.4) | 113 (49.1) | 0.811 |
| Diabetes mellitus [n (%)] | 9 (7.1) | 5 (5.7) | 14 (6.0) | 0.798 |
| Lung disease [n (%)] | 13 (1.7) | 3 (8.6) | 16 (6.9) | 0.220 |
| Liver disease [n (%)] | 17 (1.7) | 7 (13.2) | 24(10.4) | 0.704 |
| Alcohol consumption [n (%)] | 34 (12.5) | 13 (22.9) | 47 (20.4) | 0.417 |
| Biochemical indexes | | | | |
| White blood cell count (*10^9/L) | 12.4 ± 4.5 | 12.9 ± 4.7 | 12.6 ± 4.6 | 0.402 |
| Platelet count (*10^9/L) | 168.8 ± 61.4 | 163.6 ± 60.9 | 167.1 ± 61.2 | 0.549 |
| Hemoglobin level (g/L) | 111.9 ± 25.6 | 124.1 ± 141.9 | 115.9 ± 83.5 | 0.301 |
| Oxygen partial pressure (mmHg) | 172.9 ± 60.8 | 157.7 ± 51.6 | 167.9 ± 58.3 | 0.064 |
| Serum creatinine (mmol/L) | 41.5 ± 73.2 | 48.1 ± 36.7 | 43.6 ± 63.6 | 0.458 |
| Serum albumin (g/L) | 35.8 ± 23.5 | 37.3 ± 36.9 | 36.3 ± 28.5 | 0.707 |
| Serum sodium (mmol/L) | 137.5 ± 11.5 | 138.1 ± 4.8 | 137.7 ± 9.8 | 0.655 |
| Fluid records |  |  |  |  |
| Enteral nutrition (kcal/kg/48hrs) | 12.7 ± 7.6 | 36.9 ± 9.05 | 20.6 ± 13.9 | <0.001 |
| Fluid intake (ml/kg/48hrs) | 106.5 ± 43.3 | 100.3 ± 34.2 | 104.5 ± 40.6 | 0.277 |
| Fluid balance (ml/kg/48hrs) | 8.9 ± 35.7 | 3.9 ± 28.8 | 7.2 ± 33.6 | 0.289 |
| Disease severity scores | | | | |
| APACHE II score on admission  [median (IQR)] | 19.1 ± 6.0 | 18.9 ± 5.2 | 19.1 ± 5.8 | 0.774 |
| GCS on admission [median (IQR)] | 7 (5 – 11) | 7 (6 – 10) | 7 (5 – 10) | 0.652 |
| GCS at discharge [median (IQR)] | 12(8 – 14) | 12 (10 – 14) | 12 (9 – 14) | 0.160 |
| Clinical outcomes | | | | |
| Hospital-acquired pneumonia [n (%)] | 76 (55.3) | 39 (48.2) | 115 (50.0) | 0.673 |
| Other infections [n (%)] | 12 (7.1) | 3 (6.3) | 15 (6.5) | 0.281 |
| Length of ICU stay (days) | 9.3 ± 8.1 | 11.3 ± 9.8 | 9.9 ± 8.7 | 0.099 |
| Length of hospital stay (days) | 18.6 ± 8.1 | 21.2 ± 8.6 | 19.4 ± 8.3 | 0.027 |
| In-hospital mortality [n (%)] | 24 (46.4) | 2(0) | 26 (11.3) | 0.004 |

^Abbreviations: EN enteral nutrition; GCS: Glasgow coma scale; APACHE II: acute physiology and chronic health evaluation II; ICU intensive care unit; IQR, interquartile range;^

General steps of surgery procedure.

Hematoma evacuation and decompressive craniectomy

1. After general anesthesia, placing the head in the center and disinfected routinely.

2. Cut the skin, subcutaneous tissue, muscular layer and periosteum in sections along the marking line.

3. Electrocoagulation hemostasis, opening incision, stripping muscularis and periosteum, cleaning wound surface.

4. Drill three skull holes, saw the bone flap about 14cm*10cm, stop bleeding with bone wax, cut dura mater in a diffuse way, turn around, probe brain tissue, remove hematoma.

5. Stop the bleeding completely, no obvious hemorrhage is found after ascending hypertension. Reduce tension and suture dura mater, discard bone flap, and suture scalp in layers.

Ventricular drainage

1. After general anesthesia, placing the head in the center and disinfected routinely.

2. Cut the skin, subcutaneous tissue, muscular layer and periosteum in sections along the marking line.

3. Electrocoagulation hemostasis, opening incision, stripping muscularis and periosteum, cleaning wound surface.

4. Drill one skull drill, about 2*2cm in size. Use bone wax for hemostasis. The dura mater is punctured by the knife about 0.5cm. The ventricle puncture needle is punctured along the midpoint of the connection line between the external auditory meatus on both sides until the cerebrospinal fluid flows out (about 5cm).

5. The drainage tube is led out through the subcutaneous tunnel after continuing to deliver the tube for 1cm. The puncture needle is fixed to the skin and sutures the scalp in layers.
